# Extracellular vesicles in targeted drug delivery: from biological functions to surface engineering

**DOI:** 10.1186/s43556-026-00578-2

**Published:** 2026-09-16

**Authors:** Giulia Duca, Vincenza Tinnirello, Nima Rabienezhad Ganji, Riccardo Alessandro, Stefania Raimondo

**Affiliations:** 1 Department of Biomedicine, Neurosciences and Advanced Diagnostics (Bi.N.D), Section of Biology and Genetics, University of Palermo, Palermo, Italy; 2 Institute for Biomedical Research and Innovation (IRIB), National Research Council (CNR), Palermo, Italy

**Keywords:** Extracellular vesicles, Nanoparticles, Targeted drug delivery, Surface functionalization, Loading approaches, Pathological conditions

## Abstract

Achieving precise and efficient targeting represents a major challenge in drug delivery to overcome the limitations of conventional therapies. Surface functionalization of extracellular vesicles (EVs)-nanosized membranous particles released by all cell types- has emerged as a promising strategy to enhance targeted drug delivery. EVs are naturally involved in intercellular communication by transferring bioactive molecules from donor to recipient cells, and their biologically active interface confers inherent targeting properties and a favorable safety profile, making them attractive vehicles compared to synthetic nanoparticles. However, the naїve use of EVs is often limited by nonspecific biodistribution and off-target accumulation, reducing their therapeutic efficacy. To overcome these limitations, increasing efforts have focused on engineering EVs through cargo loading and surface functionalization strategies, enabling enhanced targeting and improved delivery of therapeutic molecules. In this review, we first describe the biological features of EVs, including their biogenesis, heterogeneity, and molecular composition, which underlie their potential as drug delivery systems. We then discuss the main determinants of EV-mediated drug delivery and present current engineering strategies, including cargo loading and surface functionalization approaches, highlighting their advantages and limitations. Finally, we summarize the application of engineered EVs in the treatment of several pathological conditions, including cardiovascular, neurological, autoimmune, and cancer diseases, and discuss the major challenges in clinical translation and future perspectives for EV-based targeted therapies.

## Introduction

Targeted drug delivery remains a major challenge in modern medicine, particularly in the context of complex diseases. Conventional therapeutic strategies, such as systemically administered pharmacological treatments, are widely employed in several diseases but exhibit multiple limitations, including non-specific drug localization, systemic toxicity, and reduced therapeutic efficacy [1, 2]. Moreover, conventional therapies frequently fail to consider disease heterogeneity, thereby influencing treatment response and limiting clinical outcomes [3]. The presence of biological barriers and altered tissue permeability further complicates drug delivery, often leading to overdosing and increased side effects [4, 5]. Once administered, many drugs also face challenges in maintaining their stability and bioavailability; this issue represents another flaw not limited to conventional therapies but also to the newer biological drugs, including protein and RNA interference [6]. These challenges have driven the development of new nanodrug delivery systems aimed at enhancing the targeting efficacy of treatment, thus minimizing side effects [7–9]. Over the past decades, these approaches have led to the development of nanocarriers capable of improving drug biodistribution [10], protecting therapeutic cargo from degradation [11, 12], facilitating transport across biological barriers such as the blood–brain barrier [5, 13], and enabling controlled release [14]. Among the various nanocarriers, extracellular vesicles (EVs) have attracted growing interest due to their natural origin and intrinsic role in intercellular communication [15].

**Fig. 1:**
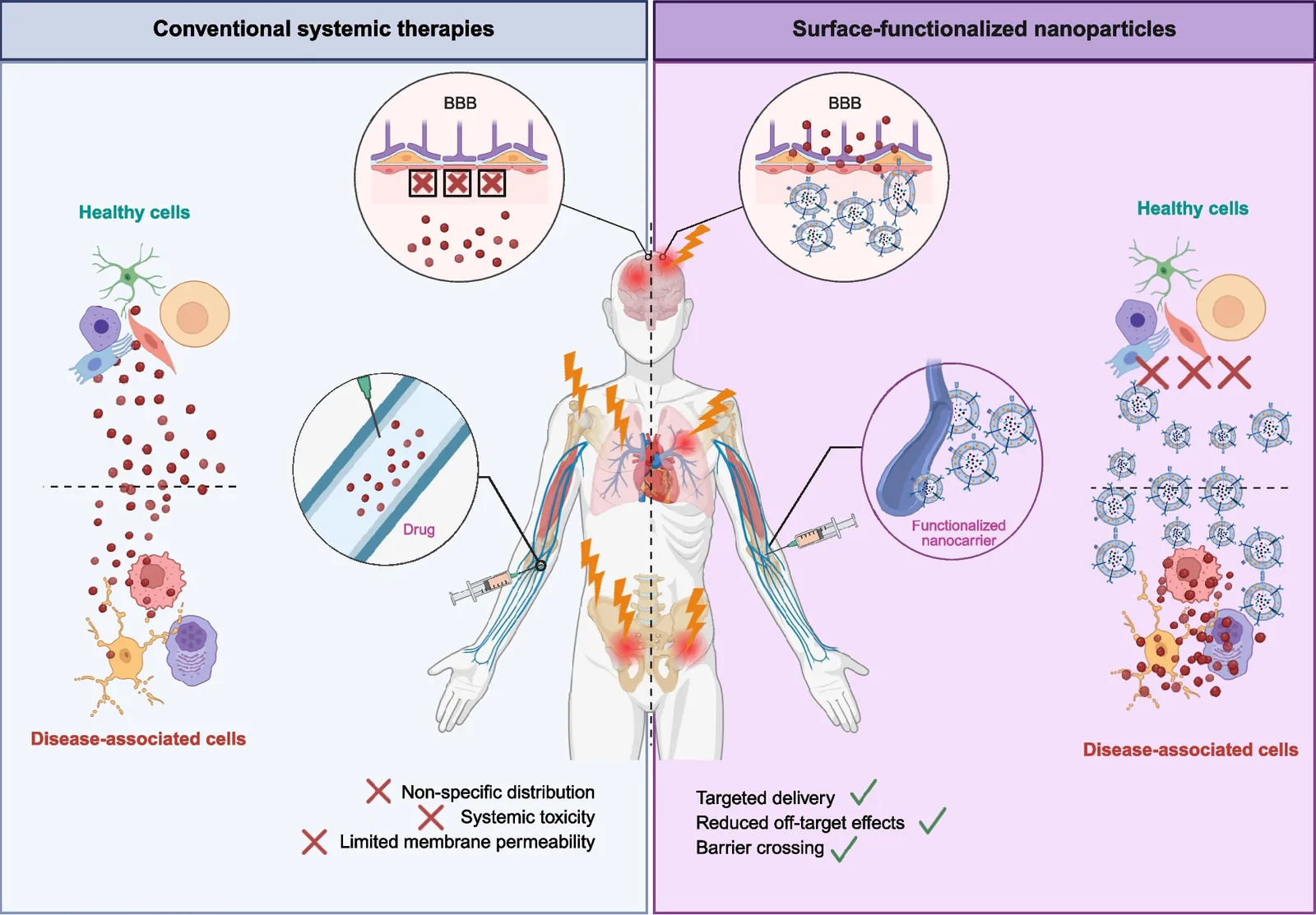
Schematic comparison between conventional systemic therapy and nanocarrier-based targeted drug delivery. Conventional treatments show a non-specific distribution and limited ability to overcome biological barriers, resulting in systemic toxicity and reduced efficacy. In contrast, surface-functionalized extracellular vesicles enable targeted delivery, barrier crossing, and controlled release at the diseased site. Created in BioRender. https://BioRender.com/c73btld. Abbreviations: BBB, Brain Blood Barrier

To further enhance their therapeutic performance, increasing efforts have focused on engineering EVs through cargo loading and surface functionalization strategies, progressively transforming EVs from naturally occurring biological carriers into targeted delivery platforms. Surface modification with bioactive molecules -such as antibodies, peptides, or specific ligands- can enhance the recognition of diseased cells, thereby improving targeting efficiency and reducing systemic toxicity and off-target effects [16]. In Fig. 1, we illustrate the limitations of conventional therapeutic strategies and highlight how nanocarrier-based targeted drug delivery, particularly through surface-functionalized extracellular vesicles, offers a promising approach to overcome these challenges.

Although numerous strategies have investigated EV biology and their therapeutic application in targeted drug delivery, the rapid evolution of EV engineering strategies highlights the need for a comprehensive and updated overview integrating EV biological properties, surface functionalization approaches, and therapeutic applications.

Here we provide a comprehensive overview of extracellular vesicles in targeted drug delivery, with particular emphasis on surface engineering strategies and their translational potential. The review is organized to progressively move from the biological basis of EVs to their application as engineered delivery platforms. First, we describe EV biology, including biogenesis, heterogeneity, molecular composition, and biological functions; second, we discuss the determinants of EV-mediated drug delivery, emphasizing the interplay between EV origin, composition, and cellular uptake; third, we present current engineering strategies, including cargo loading and surface functionalization; subsequently, we summarize therapeutic applications across major pathological conditions; finally, we discuss the main challenges that limit clinical translation and outline future perspectives.

## Extracellular vesicles: biogenesis, heterogeneity and biological functions

Extracellular vesicles (EVs) are lipid bilayer-enclosed, nano-sized particles produced and released by the cells of all living organisms, both prokaryotes and eukaryotes [17]. First identified around the 1980s, they have long been considered a disposal mechanism for cellular waste material [18]. Currently, EVs are attracting growing attention in the biomedical research field due to their recognition as key mediators of intercellular communication, playing a role in the regulation of various biological processes [19], such as immune system modulation [20, 21], tumor progression [22], and tissue regeneration [23, 24]. Importantly, EVs do not represent a uniform population, but rather a heterogeneous group of vesicles that differ in size, molecular composition, membrane organization, and biological function. This heterogeneity is a defining feature of EV biology and heterogeneity in EV membrane composition critically influences their biodistribution, cellular uptake and therapeutic potential. It arises from multiple factors, including the donor cell type and functional state, EV biogenesis pathways and environmental stimuli [25]. This intrinsic variability has contributed to the complexity of EV research, and, before 2014, there were no standardized guidelines for the study of EVs; thus, many aspects of their classification, isolation, and characterization remained a debated question. To address this issue, the International Society of Extracellular Vesicles (ISEV) published the Minimal Information for Studies of Extracellular Vesicles (MISEV) guidelines in 2014, with updates first in 2018 and then in 2023 [26]. According to MISEV 2023, the term extracellular vesicles should be used as a generic descriptor for lipidic bilayer particles released from cells. The classification of EVs suggested by the scientific consensus is based on their size, density, and molecular composition. They distinguish EVs into Small EVs (< 200 nm) and Large EVs (> 200 nm). Other terms, based on presumed biogenesis of EVs, such as Exosomes, Microvesicles, and Ectosomes, were widely used in the past to classify EVs [27]. Although still in use, these terms are discouraged unless the specific biogenesis pathway can be clearly demonstrated.

Beyond their classification, summarized in Table 1, the following subsections provide a comprehensive overview of EV biology and heterogeneity, including their biogenesis, molecular composition, and biological functions.

**Table 1:** Overview of the main extracellular vesicle subtypes, including biogenesis pathways, size range, and current MISEV2023 guidelines recommendation

EV subtype	Biogenesis pathway	Size range	Current MISEV2023 recommendation
Small EVs (sEVs)	Endosomal pathway	< 200 nm	Recommended operational term based on size
Large EVs (lEVs)	Direct budding from plasma membrane	> 200 nm	Recommended operational term based on size
Exosomes	MVB-derived ILVs	40–180 nm	Historical term; should be used only when endosomal origin is experimentally demonstrated
Microvesicles or ectosomes	Plasma membrane scission	100 nm- 1 μm	Historical term; should be used only when plasma membrane origin is experimentally demonstrated

*Abbreviations*: *MISEV* Minimal Information for Studies of Extracellular Vesicles, *EVs* extracellular vesicles, *MVB* multivesicular body, *ILVs* intraluminal vesicles

### EV biogenesis mechanisms

Exosomes, the smallest particles (approximately 40–180 nm), originate from the endosomal pathway. They are formed through inward budding of the endosomal membrane, which generates intraluminal vesicles (ILVs) within multivesicular bodies (MVBs). Upon fusion of MVBs with the plasma membrane, ILVs are subsequently released into the extracellular space as exosomes [28]. This process is mostly regulated by the ESCRT system, composed of the ESCRT-0, -I, -II and -III complexes, which are sequentially recruited to the endosomal membrane. Early ESCRT complexes recognize and sequester ubiquitinated cargo proteins [29], while ESCRT-II contributes to membrane deformation and associates with cholesterol-rich membrane domains, possibly facilitating membrane curvature and cargo sorting [30, 31]. ESCRT-III mediates the final membrane cleavage event, allowing the release of ILVs. Furthermore, the accessory protein ALIX can recruit ESCRT-III through interactions with lysobisphosphatidic acid (LBPA) in MVB membranes, further promoting vesicle formation [32].

Notably, inhibition of the ESCRT mechanism does not completely abolish exosome production, indicating the existence of ESCRT-independent pathways [33]. Among these, tetraspanins such as CD63, CD81 and CD9 play a key role in organizing specialized membrane microdomains and promoting cargo clustering during ILV formation [34, 35]. Increasing evidence suggests that tetraspanin-dependent exosome biogenesis also involves additional regulators, including ALIX, ESCRT-III, and syntenin [35, 36]. In particular, the syndecan-syntenin-ALIX axis represents an alternative mechanism in which syndecan recruits syntenin, which interacts with ALIX to promote ILV formation [36]. Although this pathway relies on ESCRT-III, it does not require ESCRT-0 or cargo ubiquitination [37]. Overall, MVB formation is a highly complex process that depends on both cargo composition and stimuli and involves redundant pathways. Once formed, MVBs can undergo lysosomal degradation or be transported to the plasma membrane, where the release of ILVs occurs. This transport is an active process driven by molecular motors and directed by small GTPases (i.e., RAB GTPases) [38]. Although the mechanisms determining MVB fate remain largely unknown, they are probably established during MVB formation [39].

Microvesicles, or ectosomes, range from 100 nm to 1 μm and display greater size heterogeneity compared to exosomes. They originate directly from the plasma membrane through outward protrusion followed by membrane cleavage [40]. Small ectosomes, whose dimensions are comparable to those of exosomes, share part of the molecular system involved in endosomal biogenesis, including ESCRT complex components and tetraspanins such as CD9, CD81, and CD82, which contribute to membrane organization and cargo selection. One of the best-characterized pathways is mediated by ARRDC1, which is responsible for the generation of arrestin domain-containing protein 1-mediated microvesicles (ARMMs) [41]. In contrast, the mechanisms underlying the formation of large ectosomes remain less clearly defined. Available evidence points to a central role of actin cytoskeleton reorganization and actomyosin contractility, which promote plasma membrane blebbing and the subsequent vesicle release. At the same time, changes in membrane lipid composition, especially in phospholipid distribution, also contribute to the process [42]

EV biogenesis pathway and the associated molecular machinery shape EV heterogeneity not only in terms of size, but also in membrane composition and biodistribution. For instance, distinct tetraspanin profiles are the result of different EV biogenesis pathways: CD63 is predominantly present on EVs originating from MVB, whereas CD81 is preferentially sorted into microvesicles [34]. These variations in membrane composition confer EVs an intrinsic cell-specific tropism, which can be exploited to direct therapeutic delivery to the desired site of action. For example, EVs displaying integrin α6 in complex with the subunits β1 and β2 preferentially target fibroblast and epithelial cells in the lung [43]. CD63-positive EVs interact specifically with neuronal and glial cells, whereas CD63-negative EVs predominantly target dendritic cells [44]. These observations highlight the importance of EV surface composition in regulating cellular recognition and uptake. Accordingly, EV membranes should not be considered merely structural barriers, but rather biologically active interfaces that critically influence EV-cell interactions and downstream signaling events [45].

### Molecular composition of EVs

EVs transport and protect from extracellular degradation a wide range of biomolecules, including proteins, lipids, nucleic acids, and metabolites. Their molecular composition results from regulated selection processes rather than from the random incorporation of cytoplasmic material and is influenced by the cell of origin, its physiological conditions, and the vesicle biogenesis pathway [42]. EV-associated proteins include both membrane-bound and intraluminal components. Among membrane proteins, previously mentioned tetraspanins, such as CD9, CD63, CD81, and CD37, are commonly present, along with integrins, adhesion molecules, and receptors involved in the recognition and uptake of vesicles. Luminal proteins include molecular chaperones such as Hsp70 and Hsp90, cytoskeletal proteins, metabolic enzymes, and proteins involved in EV formation and trafficking, including ALIX and TSG101 [46, 47]. EV membranes are also characterized by a distinctive lipid composition, enriched with cholesterol, sphingomyelin, ceramide, and phosphatidylserine, which contribute to membrane organization, vesicle stability, and signaling properties [48]. In addition, EVs transport various classes of nucleic acids, including messenger RNA (mRNA), microRNA (A), long non-coding RNA (lncRNA), circular RNA (circRNA), transfer RNA fragments, and, in some contexts, DNA fragments [49–51]. Current evidence suggests that RNA loading into EVs can occur through both passive and active mechanisms. Passive loading largely reflects the intracellular abundance of RNA species [52, 53], whereas active sorting leads to selective enrichment of specific RNA in EVs independently of their cellular levels [34]. This selection appears to depend on specific sequences or motifs present in RNAs, such as EXOmotifs, which promote loading into EVs, while other motifs (CELLmotifs) promote their retention within the cell [54]. Several RNA-binding proteins, including hnRNPA2B1 [55, 56], SYNCRIP [57], and YBX1 [58], play a central role in this context and have been implicated in the recruitment of specific RNA species into EVs through the recognition of sequences or structural motifs. Collectively, these observations show that the EV cargo composition is tightly regulated and closely linked to the originating cell and its physiological state. Indeed, comparative analyses have shown that EVs derived from different cell types exhibit distinct proteomic and membrane signatures, including tetraspanin composition [59] and selective enrichment of specific proteins and RNAs [60]. Through the transfer of proteins, lipids, and nucleic acids, EVs can modulate the behavior of the recipient cells and contribute to both normal physiological and pathological processes, highlighting their central role in intercellular communication.

### Biological functions of EVs

#### Cellular uptake and trafficking

EVs' biological activity depends not only on cargo composition but also on the ability to interact with and deliver their content to recipient cells. Following binding to target cells, EVs can be internalized through several endocytosis mechanisms, including clathrin-mediated endocytosis (CME), phagocytosis, micropinocytosis, and plasma or endosomal membrane fusion. Uptake is an active and energy-requiring process, as it is strongly reduced at low temperatures and absent in fixed cells [61]. CME is a well-characterized route in which EVs are internalized via clathrin-coated vesicles that fuse with early endosomes. Inhibition of CME by chlorpromazine significantly decreases uptake of EVs by target cells [62]. In addition, phagocytic cells, such as macrophages, take up EVs via phagocytosis; Mashayekhi et al. demonstrated that phagocytosis inhibition reduces the EV-mediated delivery of miR-181a-5p into macrophages. In the same study, inhibition of micropinocytosis produced similar effects, further supporting micropinocytosis as an additional route for internalization of EVs [63]. Importantly, different uptake mechanisms are related to distinct intracellular trafficking routes, which can affect the fate of EV cargo. Endocytic pathways often direct EVs toward endo-lysosomal compartments, where cargo degradation may occur. Therefore, endosomal escape is essential to ensure the proper release of EV molecular cargo into recipient cells. Indeed, endosomal escape is increasingly recognized as a major bottleneck in EV-mediated cargo delivery, as efficient internalization of EVs does not guarantee functional cargo release [64]. In contrast, direct membrane fusion may facilitate efficient cytosolic delivery. Once internalized, EVs can act as highly versatile carriers, capable of transporting a variety of therapeutic cargoes -proteins, nucleic acids, and small drug molecules- enabling the simultaneous delivery of multiple agents and the development of complex and multifaceted therapies [65].

#### Roles in physiological homeostasis

Healthy cells constitutively release EVs as part of physiological intercellular communication networks. Through the transfer of their cargo, EVs contribute to the regulation of multiple homeostatic processes in both local tissue environments and systemic communication. One of the first evidence of the EV biological activity came from studies on prostasomes, which demonstrated their ability to enhance sperm motility [66]. Subsequent studies demonstrated that prostasomes also contribute to immune regulation within the female reproductive tract. In particular, the presence of CD48 on prostasome membranes has been associated with modulation of NK-cell activity through interaction with the CD244 receptor, thereby reducing immune-mediated sperm clearance and prolonging sperm survival [67]. EVs derived from both human and bovine milk carry a variety of bioactive cargoes which can be delivered to immune cells and may contribute to the regulation of immune responses in the infant, thereby supporting the establishment of fetal immune tolerance [68, 69]. In the nervous system, EVs released by neurons, oligodendrocytes, and microglia participate in intercellular signaling processes involved in neuronal survival, neurite extension, synaptic activity, and myelin maintenance [70]. Similarly, intestinal epithelial cell-derived EVs contribute to tissue homeostasis and barrier function and have been proposed to participate in antigen presentation during inflammatory responses [71]. A physiological role of EVs has also emerged in the regulation of immune responses. Communication between innate and adaptive immune cells is essential for immune homeostasis, and EVs are increasingly recognized as important mediators of this crosstalk [72, 73]. EVs released by dendritic cells can carry MHC-I and MHC-II complexes capable of stimulating CD4 + and CD8 + T-cell activation, thereby promoting adaptive immune responses [74]. Conversely, EVs derived from immature dendritic cells have been associated with immunosuppressive functions, including the induction of immune tolerance and the expansion of regulatory T-cell populations [75]. Additional immunomodulatory functions have been described for EVs derived from neutrophils, macrophages, and plasma. Neutrophil-derived EVs exhibit anti-inflammatory properties by suppressing dendritic cell maturation and regulating cytokine release [76]. Macrophage-derived EVs can transfer cytokines, chemokines, antigens and regulatory RNAs to surrounding immune cells, consequently affecting both innate and adaptive reactions [77]. Plasma-derived EVs have also been associated with systemic anti-inflammatory and tolerogenic effects in several experimental models [78, 79]. Interestingly, EV-mediated regulation of physiological processes is not restricted to mammalian systems. In recent years, plant-derived nanovesicles (PDNVs) have emerged as important mediators of interspecies and cross-kingdom communication [80]. Similar to mammalian EVs, PDNVs are lipid bilayer particles capable of transporting a wide variety of bioactive molecules, including proteins, lipids, metabolites, and small RNAs, which can modulate recipient cell responses [81–83]. Increasing evidence suggests that PDNVs contribute to numerous processes such as the regulation of intestinal homeostasis [84, 85], inflammatory pathways [86–88], immune response [86], and tissue repair processes [89].

#### Roles in disease pathogenesis

Compared with EVs released under physiological conditions, disease-associated EVs often display distinct molecular signatures that reflect the altered state of the originating cells and actively contribute to disease progression through the transfer of bioactive molecules to recipient cells [90]. For this reason, EVs are increasingly recognized not only as mediators of pathogenesis but also as potential diagnostic and prognostic biomarkers [91–94]. In cancer, EVs are deeply involved in the dynamic crosstalk established between tumor cells and the surrounding microenvironment. Tumor-derived EVs can promote angiogenesis [95], extracellular matrix remodeling [96], immune evasion [97, 98], and metastatic dissemination [99] by transferring oncogenic proteins, lipids, and nucleic acids to stromal and immune cells [100, 101]. For instance, cancer-associated fibroblast-derived EVs have been shown to enhance tumor cell migration and invasiveness through activation of Wnt signaling pathways [102], while melanoma-derived EVs can reprogram bone marrow progenitor cells toward a pro-metastatic phenotype through MET signaling activation [101]. Tumor EVs are also enriched in specific miRNAs capable of modulating inflammatory and immune pathways in recipient cells, thereby contributing to the establishment of a permissive metastatic niche [103]. In addition, hypoxic conditions, commonly found within solid tumors, increase EV release and alter their cargo composition, enriching vesicles with pro-angiogenic proteins and miRNAs such as miR-210, which support tumor survival and vascularization [104, 105]. EVs have also been implicated in therapy resistance through the sequestration or transfer of drug resistance-associated molecules, including transporters and regulatory RNAs, consequently reducing therapeutic efficacy [106, 107]. Beyond oncology, EV-mediated intercellular communication has been implicated in several neurological disorders. In neurodegenerative diseases, EVs can contribute to the spread of pathogenic proteins between neural cells. EV-associated -synuclein, tau, and amyloid peptides have all been linked to the progression of Parkinson’s and Alzheimer’s diseases, suggesting that EVs may promote the spread of protein aggregates within the central nervous system [108–110]. Similarly, in prion diseases, EVs derived from infected cells can transfer pathological prion proteins to recipient cells and promote disease propagation [111]. EVs also participate in cardiovascular and inflammatory diseases. Circulating EV levels are frequently increased in patients with vascular dysfunction and myocardial infarction, where they contribute to endothelial activation, inflammation, and altered vascular homeostasis [112]. Serum levels of miR-1 and miR-133a, which regulate cardiac hyperplasia [113], are present within EVs and are significantly elevated in patients with acute myocardial infarction and angina pectoris [114]. Endothelial-derived EVs enriched with miR-143 and miR-145 have been shown to regulate smooth muscle cell phenotype and vascular remodeling during atherosclerosis progression [115]. In autoimmune disorders such as rheumatoid arthritis, EVs released from synovial fibroblasts contain inflammatory mediators and citrullinated proteins capable of sustaining chronic inflammation and modulating immune cell activation [116, 117].

**Fig. 2:**
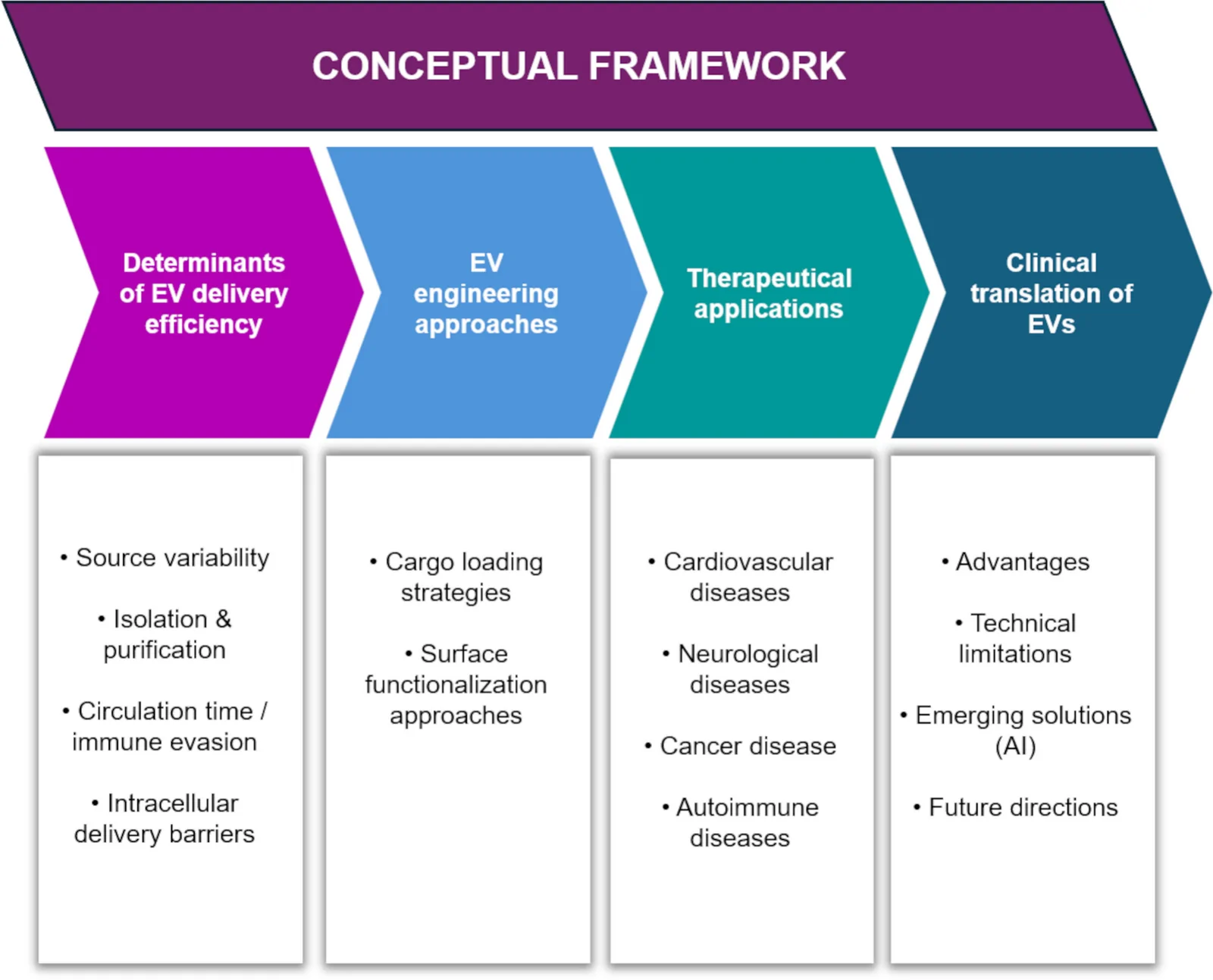
Conceptual framework of the review. Schematic overview of the rationale, key topics, and logical organization of the review, from the limitations of conventional therapies and the emergence of extracellular vesicles (EVs) as drug delivery systems to EV engineering strategies, therapeutic applications, and future translational perspectives

Collectively, these observations support the concept that EV membrane composition, cargo selection and cellular targeting are tightly interconnected processes that strongly influence EV biological activity. Building on these biological properties, this review explores the key determinants influencing EV-mediated drug delivery, the engineering strategies developed to overcome current limitations, and their applications across different pathological contexts. An overview of the conceptual framework guiding the next sections of this review is provided in Fig. 2.

## Determinants of EV-mediated drug delivery

Extracellular vesicles (EVs) have emerged as promising drug delivery systems; however, their therapeutic performance is governed by a complex interplay of biological and physicochemical factors. Compared to synthetic nanoparticles, their natural origin offers higher biocompatibility and lower immunogenicity, reducing the risk of immune reactions and toxicity [118]. Furthermore, their membrane allows easier penetration through biological barriers and promotes efficient cell-to-cell communication. However, EV-mediated delivery efficiency is dependent on the interplay between multiple determinants, including EV source, membrane and cargo composition, targeting properties, uptake routes, biodistribution, and engineering strategies, all of which collectively shape EV biological complexity and heterogeneity. Understanding these factors is essential for the rational design and clinical translation of EV-based delivery systems.

### EV source and heterogeneity

The cellular origin of Ev is a major determinant of their biological properties and therapeutic potential, as EVs partially reflect the molecular composition and biological state of their donor cells. Consequently, vesicles derived from pathological sources may retain oncogenic, inflammatory, or otherwise deleterious bioactive cargoes that could compromise their safety or influence therapeutic outcomes. Therefore, careful selection and characterization of EV-producing cells are critical to ensure the safety and efficacy of EV-based drug delivery systems. Importantly, EV variability is further compounded by the isolation and purification method; thus, an efficient isolation protocol is a critical step for downstream applications. Commonly employed methods include differential (ultra)centrifugation, density gradients, size-exclusion chromatography, fluid flow-based separation, and charge or molecular recognition-based separation methods, as described in MISEV2023 [26]. Recently, PDNVs have also emerged as an alternative natural carrier for drug delivery. Their biocompatibility, natural abundance, and scalability make them attractive for application in nanomedicine and therapeutic delivery [119, 120]. However, similar to other EVs, PDNVs exhibit marked heterogeneity and variability depending on plant source and isolation methods, representing a key challenge for standardization and clinical translation [121, 122]. Importantly, variability in EV source, culture conditions, and isolation procedures represents a major source of inconsistency across studies. Even when similar cell types or purification strategies are used, differences in donor cell state, media composition, processing steps, and analytical workflows can yield EV preparations with markedly distinct molecular profiles. This lack of standardization severely limits cross-laboratory reproducibility, complicating the interpretation of downstream functional data, including biodistribution, uptake, and efficiency of targeting strategies.

### Targeting efficiency and biodistribution

Despite their intrinsic advantages, EV therapeutic potential remains limited due to the difficulty of avoiding drug accumulation at off-target sites and the poor accumulation at the intended site of action [123]. To address these limitations, recent studies have focused on improving the delivery and the intrinsic targeting of EVs by functionalizing their surface with bioactive molecules, antibodies, receptors, or targeting ligands, enabling cell-type-dependent targeting [65].

These functionalization strategies exploit specific features of target tissues or cells, such as the overexpression of surface receptors or local microenvironmental conditions, to enhance selective delivery. To give some examples, Lai et al. developed pH-sensitive hyaluronic acid-based nanoparticles to deliver curcumin in cancer cells, realizing a controlled release system that exploits not only the altered pH of cancer cells but also the overexpression on their surface of the hyaluronic acid receptor CD44 [124]. Jayasinghe et al. functionalized the surface of extracellular vesicles from red blood cells with the targeting peptide of the CXCR4 receptor, overexpressed in many human breast cancer cells, to specifically deliver therapeutic antisense oligonucleotides [125].

However, it is important to note that the term “targeting” is widely used in a heterogeneous manner across the literature. Although improvements in therapeutic outcomes, cellular uptake, or tissue accumulation are often interpreted as evidence of active targeting, these results may not necessarily reflect true receptor-driven uptake, but rather a combination of enhanced internalization, altered biodistribution, and improved stability of cargo delivery. For example, increased accumulation in tumors or inflamed tissues may reflect enhanced vascular permeability rather than specific ligand-receptor recognition. A critical evaluation of published studies reveals that only a minority provide rigorous experimental validation to demonstrate receptor-specific targeting unequivocally. Essential controls such as receptor-blocking, competition assays, knockout of the proposed receptor, or comparison with non-functionalized EV are frequently absent. Notably, only limited studies experimentally confirm the expression of the target receptor on selected cellular models. In many cases, receptor expression is assumed based on previous reports, and enhanced cellular uptake is consequently attributed to receptor-mediated targeting without direct experimental validation. In the absence of appropriate validation, the contribution of the engineered EV–receptor interaction remains largely speculative. Another important limitation concerns the interpretation of EV biodistribution and uptake in target tissue. Many studies rely on fluorescence-based approaches without confirming that the detected signal originates from EVs. Fluorescent lipophilic dyes widely used to label EVs may generate artifacts due to dye aggregation or non-specific membrane labelling. As a result, fluorescence-based analyses often provide an indirect measure of EV distribution and do not allow the specific discrimination of vesicles from dye-associated background signals. To ensure accurate assessment of EV biodistribution and targeting, more rigorous quantitative methods -including single-particle tracking or EV-specific protein markers- are needed.

Collectively, current evidence suggests that while active targeting of EVs is achievable, it has not yet been demonstrated in a reproducible and mechanistically validated manner. Future studies should prioritize standardized assays for receptor engagement, quantitative biodistribution analyses, and cross-laboratory validation to determine whether engineered EVs can reliably achieve true targeting specificity.

### Engineering parameters affecting delivery efficiency

The therapeutic performance of engineered EVs depends on the optimization of multiple engineering parameters that collectively determine delivery efficiency. To exploit EVs as a delivery system, one central aim is to efficiently load therapeutic compounds to enrich EV cargo. In this regard, several drug-loading methods, including incubation, freeze–thaw cycles, sonication, and electroporation, may be tailored based on drug, EV source, and intended target. Efficient cargo loading represents a key parameter for the development of EV-based delivery systems; however, increasing the amount of incorporated cargo does not necessarily improve therapeutic efficacy. Excessive cargo loading may compromise EV stability or interfere with physiological interactions with recipient cells. Thus, achieving optimal loading requires an appropriate balance between cargo incorporation and preserving EV integrity and biological activity. Furthermore, another crucial aspect is assessing the effective EV-incorporated cargo; in many cases, the amount of detected cargo does not necessarily reflect the fraction encapsulated within EVs, as surface-associated or free cargo molecules may overestimate the measured signal. This aspect is not consistently addressed in many current studies, limiting reproducibility and comparability. Moreover, loading efficiency should not be considered as a direct surrogate of delivery efficiency, as therapeutic outcomes depend not only on the amount of cargo incorporated but also on cargo stability, intracellular trafficking, and effective release within recipient cells. Overall, the absence of standardized approaches for optimal loading conditions and quantification methods makes comparison across different laboratories challenging. Different studies report loading and dosing parameters using heterogeneous metrics, including particle number, total protein content, or cargo amount, limiting the comparability of results. In parallel, the performance of the functionalization strategy is determined by a combination of parameters such as ligand density, spatial orientation on EV surface, and conjugation stability. Excessive ligands on the EV surface may lead to steric hindrance reducing targeting efficiency, whereas insufficient ligands may lead to weak receptor engagement [126]. Furthermore, surface modification should be carefully optimized to enhance targeting while preserving EV biological identity, as alterations of membrane composition or surface proteins may affect vesicle stability, cellular interactions, and endogenous uptake mechanisms. Current studies often focus primarily on maximizing the loading and surface functionalization efficiency, while the impact of increased cargo incorporation and membrane modification on EV functionality is frequently less investigated. Moreover, substantial variability in reported loading efficiency and functionalization outcomes across laboratories highlights the influence of experimental variables, including EV source, culture conditions, and isolation procedures. As a result, optimal engineering parameters in an experimental system may not be generalized to other biological models. Similarly, the long-term stability of engineered EVs after modification, including potential changes during storage and administration, remains insufficiently characterized despite its relevance for clinical translation. Additionally, the effective application of EVs as a delivery system does not depend only on cargo loading and surface modification to enhance specific targeting; rather, it requires a fine balance between efficient cargo loading, optimized surface functionalization, and preservation of EV biological integrity. Therefore, future engineering approaches should move beyond the optimization of individual parameters and adopt integrated evaluation frameworks that consider cargo incorporation, EV integrity, dose definition, biodistribution, and functional delivery as interconnected determinants of therapeutic efficacy.

### Circulation and immune clearance

Importantly, surface engineering does not necessarily improve in vivo delivery, as engineered EVs may undergo biological changes following systemic administration. For instance, EVs should be rapidly cleared by the mononuclear phagocyte system, limiting their accumulation at target sites. To overcome this limitation, strategies such as PEGylation or CD47 surface engineering are needed to prolong circulation time. PEGylation is a process that covalently coats EVs with polyethylene glycol (PEG), a hydrophilic and flexible polymer. PEG shields EVs from interactions with plasma proteins and limits recognition and clearance by macrophages [127]. However, PEGylation has limitations, as the PEG coating can mask surface proteins on EVs, reducing their ability to interact with target cells. This highlights a trade-off in EV surface engineering: modifications that improve systemic stability may simultaneously impair uptake by altering membrane protein accessibility. Another strategy that overcomes this limitation involves coating with CD47, a “don’t eat me” signal that interacts with signal regulatory protein alpha (SIRPα) on macrophages, hiding EVs from phagocytosis and preventing rapid removal from circulation [128]. These approaches highlight how modulating immune recognition can enhance biodistribution without compromising targeting interactions. Both these strategies aim to prolong circulation time and systemic stability, thus increasing the probability of EVs reaching their target site. However, prolonged circulation does not necessarily translate into improved intracellular delivery, as internalized EVs may still be sequestered by endosomes. Accordingly, recent studies are focused on augmenting endosomal escape through the incorporation of membrane-disruptive elements, such as CPPs or GALA peptides, which undergo conformational changes in an acidic environment and destabilize endosomal membranes [64].

The determinants discussed in this section provide the biological and physicochemical framework that underlies EV performance as drug delivery systems and guide the development of effective engineering strategies, discussed in the following section.

## Engineering strategies to enhance EV-based drug delivery

**Fig. 3:**
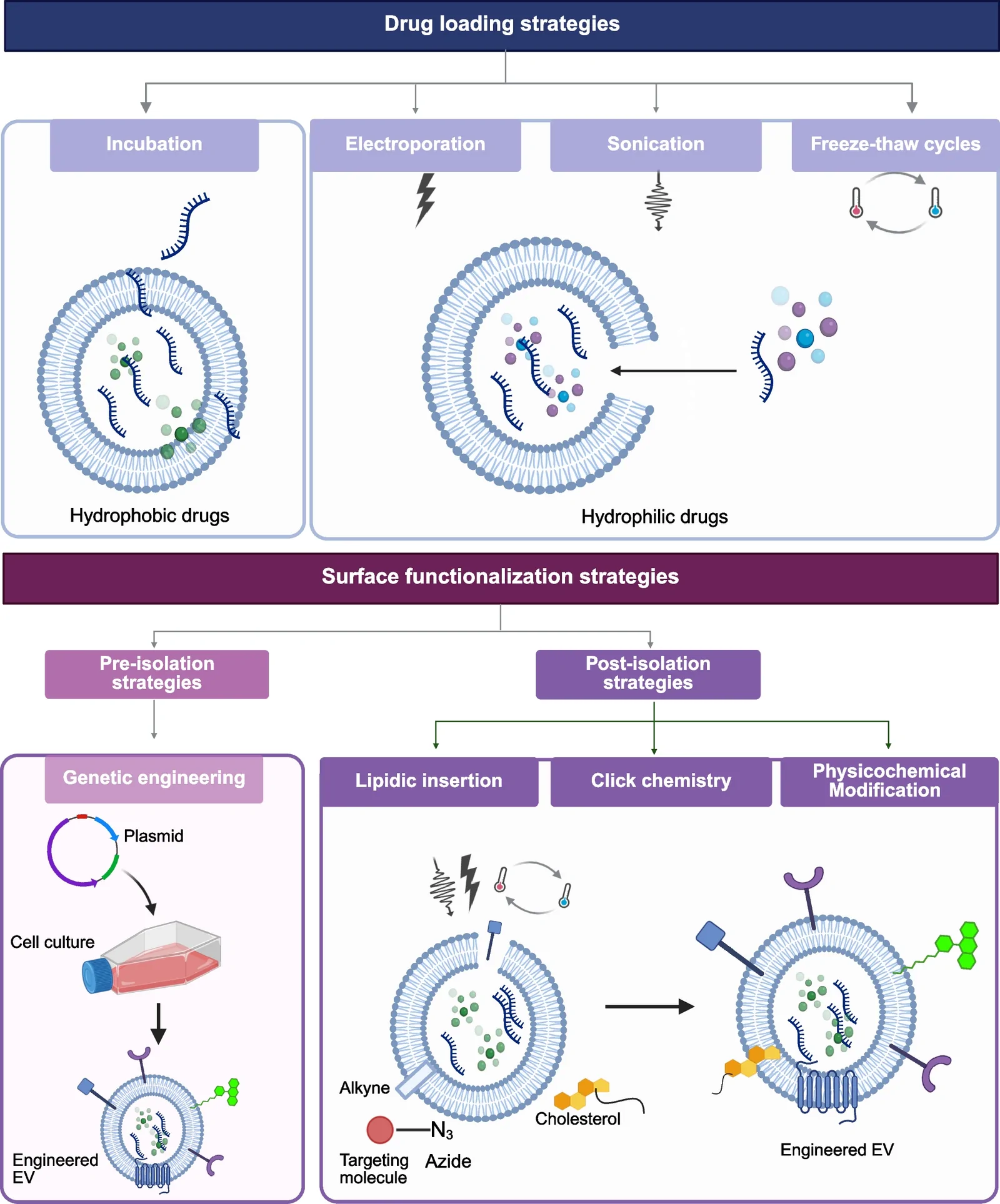
Schematic overview of extracellular vesicle (EV) engineering strategies. The figure summarizes the main approaches currently employed to engineer EVs for therapeutic applications, including both loading and surface engineering strategies. Cargo loading approaches are classified according to the physicochemical properties of the therapeutic payload, whereas surface engineering strategies are categorized into pre-isolation and post-isolation approaches performed either on EV-producing cells or directly on isolated EVs. Created in BioRender. https://BioRender.com/b8co7qf

EV engineering strategies are generally grouped into two main categories: pre-EV isolation, or endogenous, strategies, and post-EV isolation, or exogenous, strategies [34]. The pre-isolation strategies involve the genetic manipulation of the EV-producing cells or their co-incubation with the drug to directly produce intrinsically loaded and decorated EVs preserving EV integrity [129]. In contrast, in the post-isolation strategies, EVs are isolated from non-engineered cells and then modified in their cargo and in their surface content, with the advantages of not requiring the alteration of EV-producing cells but may compromise EV membrane [130]. Both approaches present their own advantages and limitations. One of the major challenges in EV engineering remains the reproducibility and efficiency of loading, as well as the maintenance of EVs' stability during manipulation to preserve their functional properties [131]. Figure 3 provides a schematic overview of the main EV engineering strategies that will be discussed in the following paragraphs.

### Cargo loading strategies

Achieving efficient loading of drugs or therapeutic molecules is crucial for employing EVs as a delivery system. Several strategies have been developed, each suited to the type of cargo to be loaded and characterized by different loading performance and impacts on EV integrity.

#### Incubation-based loading

The incubation method is a simple and non-disruptive approach that involves the co-incubation of EVs with hydrophobic therapeutic molecules, allowing their passive incorporation into EVs without affecting vesicle integrity. Due to the absence of physical or chemical disruption, this strategy is considered the least invasive loading method, particularly useful to maintain EV biological properties. However, its loading efficiency remains limited, as it strongly depends on the physicochemical properties of the therapeutic cargo. Several studies have demonstrated the potential of incubation-based approaches across different contexts. For example, curcumin, a natural compound recognized for its anti-tumoral, anti-inflammatory, and antioxidant properties, was successfully loaded into small extracellular vesicles (sEVs) derived from adipose-derived mesenchymal stem cells (ADMSCs). The curcumin-loaded vesicles significantly reduced oxidative stress and chondrocyte apoptosis in vitro and improved cartilage protection in an osteoarthritis mouse model [132]. Similarly, in a recent study, mesenchymal stem cell-derived EVs (MSC-sEVs) were loaded with bevacizumab by incubation to treat diabetic retinopathy (DR) in a rat model. The administration of these modified EVs prolonged the VEGF suppression, also extending the efficacy of bevacizumab [133]. Co-incubation was also used to load goat milk-derived EVs with the photosensitizer Chlorin e6 (Ce6) to enhance photodynamic therapy (PDT) for cancer treatment. PDT is usually limited by the poor penetration of external light into tissues. By loading Ce6 in EVs, the photosensitizer was efficiently delivered and concentrated on cancer tissue. This accumulation enhanced ROS production, improving the destruction of cancer cells [134]. Moreover, Kim et al. employed incubation to load doxorubicin (DOX) into HEK-derived EVs engineered to express SIPRα, illustrating how passive loading can be efficiently combined with surface engineering. This strategy reduced macrophage-mediated clearance from circulation, thereby increasing drug accumulation in tumoral cells, thus enhancing therapeutic outcomes [135]. Bacterial EVs derived from *Edwardsiella piscicida* (EpEVs) were loaded with the human cathelicidin antimicrobial peptide (LL37), demonstrating higher antibacterial activity compared to free LL37, upregulating the expression of immunomodulatory genes and increasing levels of ROS [136]. Collectively, these studies highlight the versatility and effectiveness of the incubation method for loading a wide range of therapeutic agents. However, compared with active loading approaches, incubation provides a lower loading efficiency since cargo incorporation depends on cargo physicochemical properties and remains limited for hydrophilic molecules due to passive diffusion across the lipidic bilayer. Notably, despite being considered a non-disruptive approach, few studies assess whether the loading process alters EV characteristics. Furthermore, differences in incubation conditions (time, temperature, adjuvants), cargo-EV ratio, purification procedures, and quantification methods complicate reproducibility across laboratories. Therefore, although incubation represents a minimally invasive loading strategy, its therapeutic efficacy remains constrained, and improvements in standardized protocols are needed to exploit its translational potential.

#### Electroporation-based loading

Electroporation is a versatile method for actively loading therapeutic cargo into pre-isolated EVs by temporarily altering the membrane permeability with an electrical field. This approach is useful for nucleic acids and other hydrophilic molecules; however, excessive electrical stress may compromise the integrity of EVs and induce cargo aggregation. To address this limitation, optimization of electroporation protocols has been explored, with particular attention to the adjustment of voltage parameters, pulse conditions, and buffer composition to minimize EV alteration and cargo aggregation [137, 138]. Using this approach, several studies successfully loaded a variety of drugs and other therapeutic molecules into the vesicles. A 2023 *Nature Nanotechnology* study used this technique to encapsulate interleukin-12 (IL-12) mRNA into EVs from HEK 293T cells. EVs were incubated in the presence of a specific electroporation buffer, with 10 μg of IL-12 mRNA, and the electroporation protocol was performed at 200 V × 5 pulses with intervals of 1 s, achieving an encapsulation efficiency of 27.6%. This approach enabled the creation of an inhalable system that inhibited lung metastasis growth by the activation of both innate and adaptive immune responses. The inhalable delivered vesicles allowed higher localization in the lungs and reduced systemic side effects [130]. Similarly, electroporation was employed to load antisense oligonucleotides (ASOs), Cas mRNA, and guide RNAs into red blood cell-derived EVs (RBCEVs), which represent an ideal source to achieve large-scale EV production. RBCEVs were electroporated with 400 pmol of anti-miR-125b ASOs at 250 V; 6 pmol of Cas9 mRNA; and 80 pmol of anti-miR-125b gRNA at 400 V. Loading efficiency of ASOs and Cas9 mRNA was measured, obtaining efficiencies of 24% and 18%, respectively. The RBCEV-mediated RNA delivery led to efficient genome editing in leukemia and breast cancer cells, both in vitro and in vivo models, with minimal cytotoxicity [139]. In immunosuppressive therapy, mesenchymal stem cell-derived small EVs (sEVs), displaying FGL1 and PD-L1 proteins, were loaded with the immunosuppressive drug FK506 via electroporation at 300 V, reaching a drug loading efficiency of 23.58%. This approach allowed the suppression of T cell activation, the reduction of CD8 + T cell density, an increase in regulatory T cells, and prolonged graft survival in a heart allograft model with minimal toxicity, demonstrating its potential as a safe and effective immunosuppressive strategy [140]. Although electroporation is one of the most efficient active loading methods, it presents numerous limitations and bottlenecks. First, following electric pulses, the EV membrane often undergoes transient structural changes; however, recovery conditions required for membrane resealing are often overlooked, potentially leading to EV fusion and aggregation. These structural changes may alter EV dimensions, promote particle loss and lower EV recovery, while reducing their internalization in target cells. In addition, cargo aggregation and artifacts may overestimate the amount of cargo effectively encapsulated within EVs. Overall, the high variability in electroporation parameters hinders reproducibility across studies and highlights the need for standardized protocols.

#### Sonication-based loading

Sonication is a strategy to disrupt the EV membrane through high-frequency sound waves, allowing an enhanced entry of drugs and therapeutic compounds inside the vesicles. For instance, Gao et al. demonstrated that by using sonication, berberine was effectively loaded into M2 macrophage-derived EVs. These modified vesicles significantly reduced the M1 marker iNOS and elevated the M2 marker CD206, reducing inflammation and facilitating the polarization of macrophages/microglia from the M1 to the M2 phenotype [141]. Zhou et al. utilized sonication to load the caspase-1 inhibitor, VX-765, into bone marrow dendritic cell-derived EVs. Caspase-1 inhibition had already shown therapeutic outcomes in a rat model of myasthenia gravis, but its use was limited due to the tissue toxicity and off-target effects. Through EV engineering, the authors achieved improved delivery of VX-765 to macrophages, enhancing its therapeutic efficacy [142]. Furthermore, Rehman et al. employed exosomal delivery to enhance the anticancer effects of temozolomide on temozolomide-resistant glioblastoma, demonstrating the potential of sonication to improve drug loading, thus overcoming drug resistance and enabling targeted drug delivery [143]. Although the sonication protocol possesses high loading efficiency, the mechanical stress generated by ultrasound may alter EV structure, promoting aggregation and thus limiting its applicability. In addition, excessive sonication can enhance thermal stress, affecting membrane protein composition and the stability of temperature-sensitive cargoes. Adjustment of acoustic parameters is required to minimize structural damage as well as the standardization of the sonication condition, enhancing comparison across studies.

#### Freeze–thaw loading

The freeze–thaw cycling approach represents an efficient strategy -more efficient than incubation, but still inferior to sonication and electroporation [144]- to load nucleic acid and small drugs into EVs, avoiding EV damage and maintaining their biological activity. This process involves repeated cycles of freezing at very low temperatures and thawing at room temperature, which can temporarily destabilize the EV membrane, offering the opportunity to load molecules into the vesicles. For example, Wu et al. developed EV-liposome hybrid nanoparticles using the freeze–thaw method, improving ALKBH5 mRNA encapsulation and mitigating the toxicity associated with liposomes, ultimately leading to significantly improved therapeutic outcomes in colorectal cancer treatment [145]. Zhuang et al. also employed the freeze–thaw cycling approach to load ribonucleoproteins (RNP) into EVs by repeating the process three times. The authors achieved approximately 30% loading efficiency, making it a good method to load EVs for drug delivery [146]. However, repeated freeze–thaw cycles may compromise EV integrity, as ice crystal formation and repeated temperature changes can alter the EV membrane and reduce the stability of temperature-sensitive cargo. Moreover, loading efficiency is strongly dependent on the number of cycles and different experimental conditions, limiting reproducibility across studies.

Collectively, these methods -summarized in Table 2- allow a versatile loading of drugs or therapeutic cargo into EVs, each with its limitations and advantages. Although active loading methods, compared to passive incubation, allow higher cargo encapsulation, these strategies may affect EV recovery yield, size distribution, and membrane organization. Furthermore, membrane destabilization and pore formation may lead to leakage of endogenous cargo, potentially losing EV intrinsic biological properties. Only a limited number of studies characterize EV cargo following exogenous incorporation, making it difficult to determine the actual drivers of observed effects. Future optimization of cargo loading approaches should consider not only cargo encapsulation efficiency but also standardized assessment of EV integrity, biological properties, and reproducibility across laboratories to improve their translational potential.

**Table 2:** EV loading methods

Loading methods	Pros and cons	EV source	Cargo	Application	Reference
Incubation	Pros: High biocompatibility, preserve EV integrity Cons: Low loading efficiency for hydrophilic and large molecules	Adipose-derived MSCs	Curcumin	Osteoarthritis	[132]
		MSCs	Bevacizumab	Diabetic retinopathy	[133]
		Goat milk-derived EVs	Chlorin e6 (Ce6)	Photodynamic therapy for cancer	[134]
		HEK 293 T cells	Doxorubicin	Cancer immunotherapy	[135]
		Edwardsiella piscicida EVs (EpEVs)	LL37 peptide	Antibacterial therapy	[136]
Electroporation	Pros: high loading of hydrophilic cargo Cons: membrane destabilization and aggregation	HEK 293 T cells	IL-12 mRNA	Inhalable immunotherapy for lung metastasis	[130]
		RBCs	ASOs, Cas9 mRNA, gRNAs	Genome editing in leukemia and breast cancer	[139]
		MSCs	FK506	Immunosoppressive therapy	[140]
Sonication	Pros: high loading efficiency Cons: membrane damage and aggregation	M2 macrophage	Berberine	M1 → M2 macrophage polarization	[141]
		Bone marrow dendritic cell	VX-765	Myasthenia gravis therapy	[142]
		Bone marrow MSC	Temozolomide	Glioblastoma therapy	[143]
Freeze–thaw cycles	Pros: moderate loading efficiency Cons: limited control over cargo encapsulation	HEK 293 T cells	ALKBH5- mRNA	Colorectal cancer therapy	[145]
		HEK 293 T cells	Ribonucleoproteins (RNPs)	Drug delivery	[146]

*Abbreviations*: *EVs* extracellular vesicles, *MSCs* mesenchymal stem cells, *HEK* human embryonic kidney, *IL-12* interleukin-12, *mRNA* messenger RNA, *RBCs* red blood cells, *ASOs* antisense oligonucleotides, *gRNA* guide RNA

### Surface functionalization strategies

Surface modification of EVs, as previously mentioned, can increase their targeting ability and enhance therapeutic efficiency while reducing drug-related side effects. Several approaches are currently under development to functionalize the EV surface.

#### Genetic engineering approaches

Genetic engineering is a pre-isolation approach to produce modified EVs spontaneously secreted by donor cells. Some of these strategies exploit EV membrane proteins—Lamp2b, CD81, CD63, and CD9-as anchors to display targeting molecules. In this process, donor cells are transfected with plasmids encoding the chosen membrane protein fused to a ligand for specific targeting. Among the membrane proteins, Lamp2b is one of the most used, due to its high expression in the EV membrane and its tolerance for peptide fusion. One of the earliest pieces of evidence of Lamp2b-based EV surface functionalization was reported by Alvarez-Erviti et al. In this study, donor dendritic cells were genetically engineered to express Lamp2b fused to a neuron-specific rabies virus glycoprotein (RVG) peptide, which specifically binds the acetylcholine receptor. Furthermore, functionalized EVs were loaded with siRNA targeting BACE1, a key enzyme involved in Alzheimer’s disease pathogenesis. Following systemic administration, engineered EVs achieved efficient and selective siRNA delivery to neuronal cells across the BBB, resulting in a significant reduction in BACE1 gene expression. Subsequently, several studies have expanded this strategy to different models [147]. For instance, Liang et al. created an EVs-based delivery system to transport miR-140 into chondrocytes across the nonvascular extracellular matrix of cartilage. They fused Lamp2B with a chondrocyte-affinity peptide (CAP) to target chondrocytes, offering a promising therapeutic approach for osteoarthritis. These CAP-EVs, in contrast to non-tagged EVs, showed enhanced uptake by chondrocytes. Moreover, CAP-EVs could efficiently encapsulate miR-140, reducing cartilage degradation in vivo, supporting their therapeutic applicability to alleviate osteoarthritis progression [148]. Zhao et al. decreased chemotherapy resistance in ovarian cancer. The authors aimed to specifically target and block angiogenesis by EVs to normalize vascular function. EVs were engineered to display an integrin-targeting peptide (RGD) on their surface through Lamp2b and loaded by electroporation with miR-484, which is downregulated in cancer cells and angiogenic endothelial cells. The modified EVs were enriched in the tumor mass, improved vascular normalization and sensitivity to chemotherapy, and prolonged the survival of mice after the treatment [149]. Bellavia et al. engineered EVs derived from HEK293T cells to express Lamp2B fused to Interleukin-3 (IL-3) to target chronic and acute myeloid leukaemia (CML and AML). The IL-3 receptor was recognized as overexpressed in CML and AML cells, while expressed at low levels in normal hematopoietic stem cells. Engineered EVs were loaded with Imatinib or BCR-ABL siRNA, enabling their specific delivery to tumor cells and leading to a reduction in tumor size in vitro and slower tumor growth in vivo [150]. In another study, 293T cells were transfected with the gene fusion between CD9 and Apo-A1 to selectively target hepatocarcinoma cells, reducing cell proliferation thanks to the delivery of miR-26a. ApoA1 is a well-known target of the SR-B1 receptor, highly expressed on the surface of many cancer cells, including HepG2. The CD9-ApoA1 EVs showed increased internalization by hepatocarcinoma cells, and the delivery of miR-26a arrested the cell cycle and reduced cell proliferation [151]. Despite allowing extensive surface modification, the application of genetic engineering approaches is still limited by technical complexity and translational feasibility. This strategy generally preserves EV integrity better than post-isolation approaches, while enabling stable and homogeneous ligand display. However, engineering donor cells is time-consuming, requires efficient transfection, and may alter cellular physiology, affecting EV composition and biological activity. Moreover, expression levels are dependent on the donor cell type, posing challenges for reproducibility and clinical translation.

#### Lipid insertion strategies

Lipid insertion is a post-isolation strategy in which lipids can be passively incorporated into the EV membrane, exploiting the hydrophobic affinity with the lipidic bilayer. In this approach, lipids can be conjugated with bioactive molecules and then incorporated into the EV, conferring specific targeting. For instance, DSPE-PEG is a lipid used to support the insertion of targeting molecules into the EV surface. Wu et al. used DSPE-PEG to decorate EV with angiopep-2, increasing BBB penetration and the targeting ability of docetaxel (DTX)-loaded EV against glioblastoma. This modification enhanced brain delivery, increasing the DTX concentration in the tumor area, also reducing the side effects of chemotherapy [152]. Cholesterol is also widely used; for example, Wang et al. modified the EV surface to conjugate AS1411, which binds nucleolin, highly expressed on the membrane of breast cancer cells. These vesicles were loaded with fluorescent let-7 miRNA to confirm delivery specifically to breast cancer cells. The cholesterol-anchored AS1411 enhanced EV internalization in tumor cells, improving the anti-proliferative effects of their cargo in tumor growth [153]. Additionally, Pi et al. conjugated three classes of ligands through a cholesterol chain. First, folate, whose receptor is overexpressed in many cancers of epithelial origin, such as colorectal cancer, was conjugated, followed by PSMA and EGFR aptamer, respectively, to target prostate cancer and breast cancer. The authors loaded these modified EVs with survivin siRNA, evaluating in vivo the inhibition efficacy in the three cancer models. Their multiple-aptamer strategy demonstrated efficient and selective binding to each tumor type [154]. Compared with genetic engineering, lipidic insertion is technically simpler and faster, making it attractive for scalable manufacturing. However, lipid insertion stability relies on hydrophobic interaction, which may lead to ligand dissociation in biological fluids. Furthermore, since excessive lipid incorporation may alter membrane composition, affecting EV uptake and biodistribution, optimization is required to balance preservation of EV properties and targeting efficiency.

#### Click chemistry approaches

Click chemistry is one of the most robust post-isolation approaches that allows a stable and efficient linkage of targeting molecules to the EV surface. In particular, the process employs two separate chemical reactions to covalently conjugate both the EV membrane and the targeting molecule, with complementary reactive groups that can react with each other [155]. Mun et al. used click chemistry to improve cardiac targeting by conjugating the T peptide to EVs. DBCO-NSH was first incorporated into amino groups on the EV surface, and then the T peptide, modified with azido, reacted with DBCO, establishing a covalent conjugation. These modified EVs exhibited enhanced homing to injured myocardium and improved cardiac function in a myocardial infarction model [156]. Similarly, Tian et al., through a DBCO-NSH reaction, introduced the reactive DBCO groups on the EV membrane, which reacted with an azide-modified RGDyK peptide. The engineered cRGD-Exo increased delivery to the cerebral vascular endothelium after ischemia, enhancing the accumulation in ischemic brain regions. Furthermore, curcumin has been loaded into the modified Exo, leading to a strong suppression of inflammation, cellular apoptosis, and neuroprotection [157]. Despite its advantages, click chemistry presents mechanistic limitations; first, the introduction of an azide or alkyne group may perturb lipidic organization, affecting EV stability. Moreover, reaction efficiency is strongly dependent on the density and the accessibility of reactive groups, leading to variability across final products. In addition, click chemistry requires controlled reaction conditions, specialized reagents, and rigorous purification steps to remove residual catalysts or unreacted components, all of which limit scalability and clinical translation.

#### Physicochemical modification strategies

Physical surface engineering methods include electroporation, sonication, and freeze–thaw cycles. Even though these approaches are mainly employed to load cargo into the EVs by temporarily disrupting and permeabilizing their membranes, they offer the possibility to insert ligands that modify the EV surface [158]. For instance, sonication was used to simultaneously load DOX and pH-responsive molecules to specifically target cancer tissue. Drug delivery systems responsive to pH are emerging as a potent tool to selectively transport antitumor drugs specifically to cancer cells, exploiting the acidic environment where pH is lower compared to normal tissue. In this study, the authors engineered EVs with a pH-responsive molecule consisting of hyaluronic acid grafted with 3-(diethylamino) propylamine (HDEA), which bound CD44 receptors on HCT-116 tumor cells and efficiently released DOX in response to the acidic pH [159]. Cheng et al. exploited freeze–thaw cycles to incorporate a chimeric peptide (ChiP) on the EVs' surface. The ChiP consisted of an alkyl chain for EV engineering, a photosensitizer, and a nuclear localization peptide (NLP), improving nucleus-targeted PTD therapy, consequently improving the inhibition of tumor growth [160]. Since these approaches were primarily developed as loading techniques, physicochemical modifications used for EV surface engineering remain the least specific strategies and share many of the limitations already discussed for cargo loading. Nevertheless, they benefit from enabling simultaneous cargo loading and surface functionalization within a single workflow.

Overall, many studies provide limited quantitative characterization of EV functionalization. In particular, the density of targeting ligand is rarely measured, making it challenging to correlate ligand display with targeting efficiency. Similarly, although surface modification may alter EV size, membrane integrity, and surface charge, a comprehensive post-engineering characterization is frequently lacking. Furthermore, while many studies compare engineered EVs with unmodified ones, fewer evaluate experimental controls such as free targeting ligand and free therapeutic cargo, which are essential to distinguish the contribution of EV-mediated delivery. Addressing these methodological limitations through standardized characterization and rigorous experimental design will be essential to improve reproducibility and clinical translation of engineered EVs.

A summary of the surface functionalization approaches discussed in the previous section is provided in Table 3.

**Table 3:** EV surface engineering methods

Surface modification technique	EV source	Surface anchor/chemistry	Targeting ligand	Cargo	Application	Reference
Genetic engineering	Dendritic cells	Lamp2b	RVG	siRNA BACE1	Alzheimer's disease	[147]
	Dendritic cells	Lamp2b	Chondrocyte-affinity peptide (CAP)	miR-140	Osteoarthritis therapy	[148]
	HEK293T cells	Lamp2b	RGD peptide	miR-484	Ovarian cancer	[149]
	HEK293T cells	Lamp2b	IL-3	Imatinib/BCR-ABL siRNA	CML and AML	[150]
	HEK293T cells	CD9	ApoA1	miR-26a	Hepatocellular carcinoma	[151]
Lipid insertion	MSC	DSPE-PEG	Angiopep-2	Docetaxel (DTX)	Glioblastoma	[152]
	Bone marrow dendritic cells	Cholesterol	AS1411 aptamer	let-7 miR	Breast cancer	[153]
	HEK293T cells	Cholesterol	Folate/PSMA aptamer/EGFR aptamer	Survivin siRNA	Colorectal, prostate, and breast cancer	[154]
Click chemistry	Patients blood	DBCO–azide reaction	T peptide	CRISPR-Cas9 RNP	Myocardial infarction	[156]
	Bone marrow MSC	DBCO–azide reaction	RGDyK peptide	Curcumin	Ischemic stroke	[157]
Physical methods (sonication)	Macrophage (RAW 264.7)	Membrane disruption	HA–HDEA (pH-responsive)	Doxorubicin (DOX)	Colorectal cancer	[159]
Physical methods (freeze–thaw)	Mice blood	Membrane destabilization	Chimeric peptide (ChiP)	—	Cancer therapy	[160]

*Abbreviations*: *EVs* extracellular vesicles, *Lamp2b* Lysosome-associated membrane protein 2b, *RVG* rabies virus glycoprotein, *siRNA* small interfering RNA, *miR* micro RNA, *HEK* human embryonic kidney, *RGD* integrin-targeting peptide, *IL3* interleukin 3, *CML* chronic myeloid, *AML* acute myeloid leukaemia, *MSC* mesenchymal stem cell, *DSPE-PEG* 1,2-distearoyl-sn-glycero-3-phosphoethanolamine-poly(ethylene glycol), *PSMA* Prostate-Specific Membrane Antigen, *EGFR* Epidermal Growth Factor Receptor, *DBCO* dibenzocyclooctyne, *CRISPR* Clustered Regularly Interspaced Short Palindromic Repeats, *RNP* ribonucleoprotein, *HA-HDEA* hyaluronic acid grafted with 3-(diethylamino)propylamine

As summarized in Table 4, despite the extensive literature, no single EV modification strategy currently demonstrated superiority among the considered parameters. Therefore, since an universal approach has not emerged, its selection should be led by the intended therapeutic application, production feasibility and clinical translation.

**Table 4:** Comparative overview of major EV engineering strategies

Strategy	Loading efficiency	Preservation of EV integrity	Scalability	Translational potential
Genetic engineering	Moderate-High	High	Low	Moderate
Lipid insertion	Moderate	Moderate	High	High
Click chemistry	Moderate	Moderate	Moderate-Low	Moderate
Electroporation	High	Low	Moderate	Moderate
Sonication	High	Low	Moderate	Low
Freeze–thaw cycles	Moderate	Moderate	High	Moderate
Incubation	Low	High	High	Moderate

## Therapeutic applications of engineered extracellular vesicles

An increasing number of studies are exploring the application of engineered EVs in several pathological conditions. Although similar EV engineering strategies are frequently used across different applications, the design and implementation of engineered EVs are tailored to the specific pathological context and therapeutic goals. Accordingly, this section discusses the current application of engineered EVs in major disease areas, highlighting representative preclinical advances.

### Cardiovascular diseases

Cardiovascular diseases (CVD) include several pathological conditions, like myocardial infarction, coronary heart disease, and pulmonary embolism. Their development and progression are driven by multiple risk factors such as obesity, hypertension, atherosclerosis, and dyslipidaemia [161]. In CVD, the limited regenerative ability of cardiomyocytes hampers the recovery of damaged myocardium following ischemic events. Changes in protein expression within ischemic tissue can be exploited as molecular targets to selectively deliver therapeutic cargoes to the infarcted area [162]. In this context, extracellular vesicles represent one of the most promising strategies to deliver cardioprotective molecules to ameliorate CVD treatment, by reducing cell death and inflammation [163]. Chen et al. engineered bone marrow mesenchymal stem cell-derived EVs to reduce inflammation in atherosclerosis progression. Cells were first transfected with the recombinant silent information regulator 2–related enzyme 1 (SIRT1) adenoviruses to generate EVs overexpressing SIRT1. Then, to enhance their targeting, EVs were modified by cholesterol insertion of VEGF to specifically target vascular endothelial cells. SIRT1 regulated the activation of Nrf2 and reduced oxidative stress in target cells, thereby reversing endothelial-mesenchymal transition and reducing the formation of atherosclerotic plaques [164]. While the previous study focused on improving targeting within the endothelial compartment, subsequent approaches have shifted to peptide-mediated targeting to improve EV accumulation within ischemic myocardium. For example, Wang et al. generated BM-MSC-derived EVs expressing an ischemic myocardium targeting peptide (IMTP). The IMTP (CSTSMLKAC), discovered via an *in vivo* phage display technique, is a peptide able to selectively target the ischemic heart compared to normal tissue. Accordingly, they observed an increased EV uptake into a hypoxic cardiomyoblast cell line in vitro, and an enhanced distribution in the heart tissue of infarcted mice [165]. Building on the same targeting strategies, Tong et al. developed an EV-based delivery system in which milk-derived EVs were first functionalized with IMTP and then encapsulated with miR-30d. Authors demonstrated that EVs loaded with miR-30d alleviated cardiac hypertrophy and inflammation in murine models by targeting the GRK5-mediated chemokine signaling pathway [166]. Beyond IMTP, additional peptides have been investigated to improve EV localization in the ischemic myocardium; in this context, Vandergriff et al. conjugated EVs with the cardiac homing peptide, obtaining an enhanced retention of modified EVs in the injured tissue and promoting cardiac regeneration [167]. Similarly, to facilitate the recovery of cardiac function in infarcted myocardium, Mun et al. first modified the EV surface with cardiac-targeting peptide T to target cardiac tissue, and then loaded EVs with CRISPR-Cas9 RNP, achieving cardiac genome editing and reducing myocardial damage [156]. Collectively, these studies highlight the potential of engineered EVs to enhance cardiac-specific delivery and tissue repair following ischemic injury. Current evidence suggests that peptide-mediated surface functionalization represents the most explored strategy to improve myocardial targeting. However, direct comparison among different targeting peptides is still lacking, making it difficult to identify the optimal targeting strategy. Future studies should perform a comparison of targeting ligands while evaluating biodistribution and long-term cardiac function. Notably, differences in therapeutic outcomes not only depend on improved cardiac homing but also on the intrinsic regenerative properties of EV source and the nature of the encapsulated cargo. In addition, combination with other approaches may provide complementary advantages depending on the pathological context.

### Neurological disorders

Neurological diseases include both chronic neurodegenerative conditions and ischemic brain injuries. The protection provided by the BBB significantly limits drug delivery to the brain, making the treatment of neurological diseases one of the greatest challenges [168]. EVs, due to their capability to cross the BBB, can be exploited to develop delivery vehicles targeting neurological disorders, aiming to promote neuronal regeneration and cerebral restoration [169]. The functionalization of the extracellular vesicle surface may enhance their specific targeting, hence their therapeutic effects [170].

#### Neurodegenerative diseases

Neurodegenerative diseases are a complex group of pathologies, widespread worldwide, characterized by the accumulation and aggregation of misfolded proteins, causing cell death and consequently neuronal loss and brain damage [171]. Alzheimer’s and Parkinson’s diseases are the most common neurodegenerative disorders. Targeted EV-based strategies have been developed to address misfolded proteins in Alzheimer’s disease. For instance, Iyaswamy et al. genetically engineered vesicles derived from hippocampal neuronal cells with Fe65 protein to target specifically cells with accumulation of amyloid-β precursor protein (APP) typical in Alzheimer's disease. Then the researchers loaded functionalized EVs with Corynoxine-B (an autophagy stimulator), inducing cellular death of neurons overexpressing APP in murine models [172]. Other recent studies aimed to enhance EV accumulation within the brain through neuron-targeting ligands, such as RVG, which promotes neuronal targeting through interaction with acetylcholine receptors. For instance, mesenchymal stem cell-derived EVs were modified by Cui et al. with RVG to specifically target the brain of an Alzheimer’s murine model, reducing inflammation and ameliorating cognitive function [173]. In the context of Parkinson’s disease, Ren et al. generated RVG-functionalized EVs loaded with DNA aptamers targeting α-synuclein aggregates, the main components of Lewy bodies. These engineered vesicles efficiently deliver the aptamers to neuronal cells and ameliorated behavioural deficits in the mouse brain [174]. A different approach was used by Sul et al., which engineered EV surfaces with dopamine, enhancing the dopaminergic neuronal uptake of EVs and stimulating autophagy in a Parkinson's induced model [175].

#### Ischemic brain disorders

Ischemic brain diseases are characterized by a reduction or interruption of blood flow to the brain, leading to neuronal injury and cell death. These pathologies include both acute events and chronic conditions and are usually due to inflammation, oxidative stress, and loss of normal neuronal function.

Tian et al., using click chemistry, functionalized the surface of EVs with the c(RGDyK) peptide, which recognizes integrin in cells after ischemic stroke, to treat a mouse model of ischemic brain. They observed a strong reduction of the inflammatory response and apoptosis when injured mice were treated with functionalized vesicles loaded with curcumin [157]. Shi et al. modified EVs derived from M2-microglia with polydopamine (PDA) and the neuronal targeting peptide rabies virus glycoprotein peptide 29 (RVG29) to target the stroke mice's brain. A PDA coating was first realized under alkaline conditions, enabling the formation of an adhesive layer on the EV surface that served as a bridge for the covalent attachment of RVG29. This peptide has shown neuronal targeting by binding the nicotinic acetylcholine receptor, enhancing neuronal uptake in vitro. The modified EVs were administered to mice with a stroke event, resulting in a reduction of apoptotic and degenerative neurons, suggesting their neuroprotective effects. These effects were related to the miRNA EV content, particularly enriched in miR-221-3p and miR-423-3p, both involved in anti-apoptotic processes, via the p38/ERK signaling pathways [176].

Collectively, these findings indicate that enhancing BBB penetration and neuronal targeting represents a common strategy to improve the efficacy of EV-based therapies in neurological disorders. Among the different approaches investigated, RVG-based functionalization currently represents one of the most used strategies due to its ability to promote neuronal interactions; however, its superiority over alternative targeting approaches has not been demonstrated. Nevertheless, the wide variability in targeting strategies employed across studies makes it challenging to determine which strategy provides the greatest therapeutic benefit. In addition, long-term recovery and safety evaluation remain insufficiently investigated. These limitations need to be addressed to consider the potential of engineered-EV for clinical translation.

### Cancer

Cancer is one of the major causes of death worldwide, with an increasing incidence over the last decade. It arises from alterations in genes involved in cell growth, immune evasion, differentiation, and survival, leading to the formation of abnormal cell masses [177]. Current cancer treatments often involve significant side effects due to the non-specific targeting of the administered drug. One of the purposes of cancer research is to develop targeted drug delivery systems to solve the flaws of existing treatments. The multiple alterations and modifications in cancer cells often lead to the overexpression of molecules and receptors that promote cancer proliferation and tissue invasion. EVs can be functionalized on their surface with peptides, antibodies, or proteins to bind these overexpressed molecules, enabling the specific targeting of cancer cells [178]. Several studies have explored these strategies, highlighting the versatility of EV-based delivery systems.

To enhance the anti-cancer potential of T-cells, Jung et al. generated T-cell-derived EVs coated with interleukin-2 (IL2) by transfecting producing cells with a lentiviral vector encoding IL2. These EVs reinforced the CD8^+^ T cells' activity and reduced the expression level of PDL1 in melanoma cells, effects mediated by miRNAs (miR-181a-3p and miR-223-3p) whose expression was increased by autocrine effects of IL2 [179]. While Jung et al. focused on enhancing the immune response via T-cell-derived EVs, other studies aimed to directly target tumor cells carrying therapeutic molecules. For instance, based on the knowledge that pancreatic cancer cells overexpress integrin α, Zouh et al. genetically modified the surface of EVs derived from bone marrow mesenchymal stem cells (BMCSs) with the targeting peptide of integrin α. This modification aimed to enhance the delivery of EVs to cancer cells both in vitro and in vivo. Furthermore, they transfected the BMSCs to overexpress miR-148a-3p within the EVs, reducing tumor growth by inactivating the downstream TGFβ/SMAD pathway [180]. Beyond direct tumor targeting, engineered EVs have also been explored to modulate stromal components of the tumor microenvironment. BMSC EVs were modified with the targeting peptide of integrin α and then loaded with miR-138-5p and the anti-fibrotic agent pirfenidone (PFD) to reprogram cancer-associated fibroblasts (CAFs). miR-138-5p inhibited the TGFβ signaling pathway and, in combination with PFD, promoted pancreatic cancer suppression [181]. In glioblastoma treatment, heme oxygenase 1 (HMOX1) was identified as a membrane cancer marker for GBM. Rehman et al. decorated EVs derived from bone marrow mesenchymal stem cells with a HMOX1-specific peptide (HSSP) to deliver temozolomide (TMZ) and STAT3-specific siRNA for GBM treatment [143]. In addition to mammalian-derived EVs, plant-derived vesicles have also been explored as natural nanocarriers for cancer drug delivery [182]. Compared to their mammalian counterparts, plant-derived EVs offer several advantages, including lower production costs and high scalability, making them particularly attractive for translational applications. For example, Long et al. isolated and functionalized orange-derived extracellular vesicles to specifically target ovarian cancer. They engineered the EV membrane, thereby enhancing their accumulation and penetration ability in cancer cells, reducing growth in ovarian cancer [183]. In lung cancer treatment, grapefruit EVs (GEV), loaded with pterostilbene (PS), were functionalized to enhance tumor targeting, with lactoferrin (LF/PS-GEV), an iron-binding glycoprotein whose receptor is overexpressed on lung tumor cells. Sonication was used to load PS -a bioactive phytochemical- into GEV, and electrostatic interactions were used to coat GEV with LF. In in vitro models, the developed vesicles were strongly internalized by lung cancer cells (A549), resulting in reduced cell viability and cell migration. In vivo treatment with LF/PS-GEVs significantly decreased tumor growth, downregulating pro-angiogenic and proliferative markers (VEGF and cyclin D) while apoptotic markers were upregulated [184].

Despite substantial progress, the clinical translation of tumor-targeted EVs remains challenged by tumor heterogeneity and variability in receptor expression across patients. These factors largely influence targeting performance, shaping whether a ligand will be effective across different tumor models. In addition, the wide variety of targeting ligands, EV sources, and therapeutic cargos employed across studies makes direct comparison particularly challenging.

### Autoimmune diseases

Autoimmune diseases are pathological conditions in which the immune system loses tolerance, recognizing healthy cells and tissues as non-self, leading to inflammation and tissue damage. The most common autoimmune diseases include multiple sclerosis (MS), rheumatoid arthritis, systemic lupus erythematosus, psoriasis, and others. Selective delivery of immunomodulatory agents to inflamed tissues remains a major challenge in autoimmune diseases, where systemic immunosuppression often leads to severe side effects and limited therapeutic specificity. Thanks to their ability to modulate immune response, EVs represent a promising approach to target inflamed tissue [185, 186]. Shamili et al. covalently conjugated LJM-3064 aptamer to the surface of mesenchymal stem cells-derived EVs as a delivery system against MS. LJM-3064 is a 40 nt single-stranded aptamer that, due to its guanosine-rich sequence, forms specific 3D structures, enabling high myelin affinity. This aptamer demonstrated immune modulation activity and remyelination induction, acting both as a targeting ligand and a therapeutic agent. The authors observed increased accumulation of modified EVs in demyelinated regions, leading to reduced inflammation and demyelination, and improved recovery in animal models [187]. Based on click chemistry and glycoengineering, Lim et al. produced PEGylated hyaluronic acid-modified EVs, capable of binding cells expressing high levels of CD44, accumulating in models with rheumatoid arthritis. These modifications also improved EV stability in circulation, enhancing their therapeutic efficiency [188]. Huang et al. loaded GEVs with the immunosuppressor CX5461; to achieve specific targeting, GEVs were fused with mesenchymal stem cell-derived EVs previously engineered to express CCR6, a receptor whose ligand is overexpressed in inflamed tissues. These modifications allowed the vesicles to specifically target the inflamed sites and reduce inflammation in psoriasis and atopic dermatitis disease models [189]. Although engineered EVs have shown encouraging immunomodulatory effects across different autoimmune disease models, current evidence remains limited to a relatively small number of preclinical studies.

**Fig. 4:**
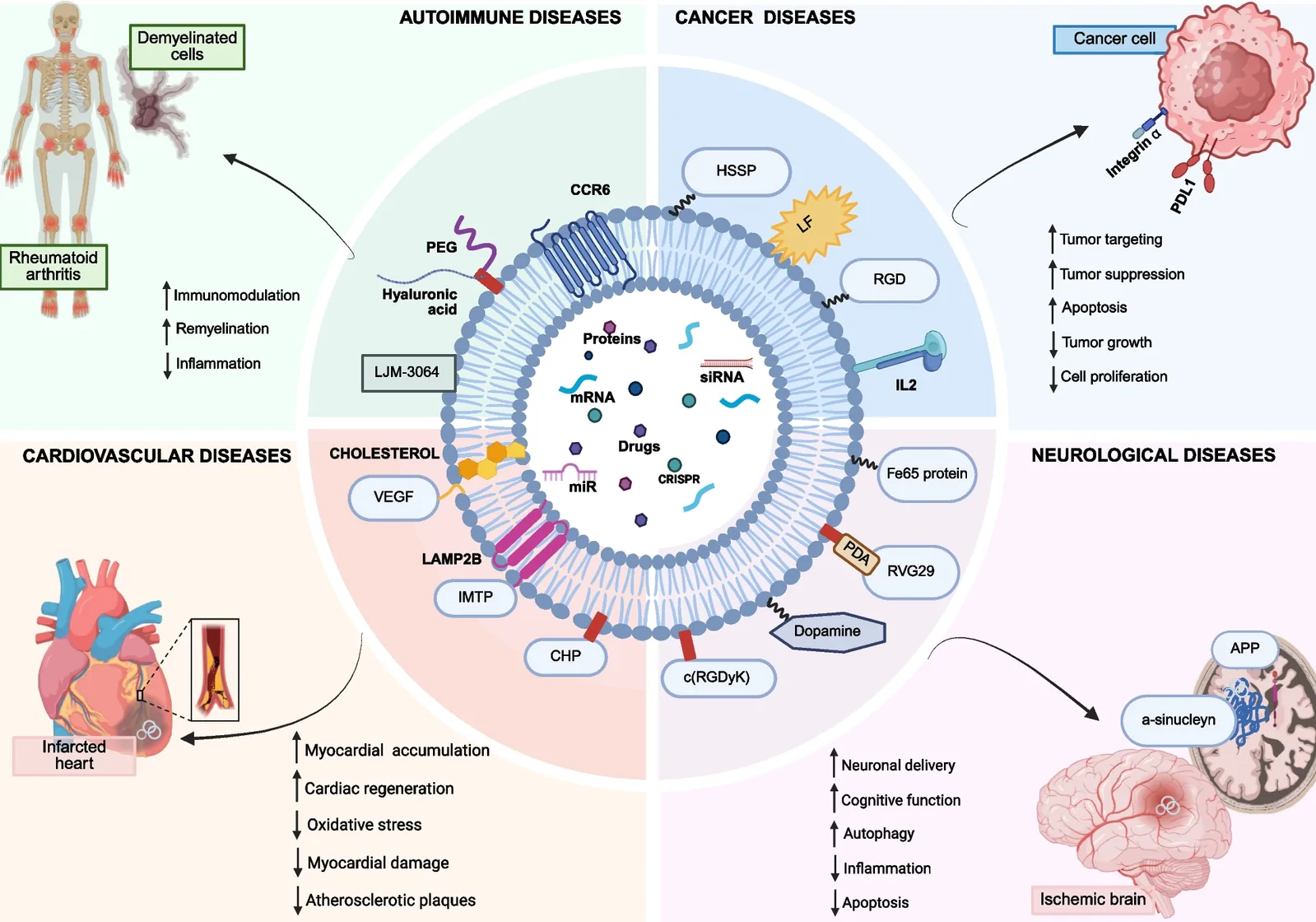
Overview of extracellular vesicle surface functionalization strategies for disease-specific targeting. EVs can be engineered with peptides, antibodies, or proteins to selectively target cardiovascular, neurological, oncological, and autoimmune diseases by exploiting disease-associated molecular signatures. Created in BioRender. https://BioRender.com/ltcif8f. Abbreviations: VEGF, Vascular Endothelial Growth Factor; IMTP, Ischemic Myocardium Targeting Peptide; Lamp2b, Lysosome-associated membrane protein 2b; CHP, Cardiac Homing Peptide; RGD, integrins targeting peptide; RVG, Rabies Viral Glycoprotein; PDA, Polydopamine; APP, amyloid-β precursor protein; IL2, Interleukin 2; LF, Lactoferrin; HSSP, HMOX1-specific peptide; CCR6, Chemokin Receptor Type 6; PEG, Polyethylene Glycol; siRNA, small interfering RNA; miR, microRNA

Collectively, these studies support the potential of engineered EVs as targeted therapeutic platforms. Across different pathological contexts, surface engineering improves tissue-specific accumulation and therapeutic efficacy; however, the magnitude of these benefits differs depending on several factors, including EV source, engineering strategy, target receptor expression, and disease microenvironment. Despite promising preclinical outcomes, several translational challenges remain unresolved, and further investigations are needed to evaluate biodistribution and long-term safety, repeated-dose immunogenicity, and the reproducibility of EV engineering strategies in clinically relevant settings. Furthermore, large-scale manufacturing, quantitative assessment of targeting efficiency, and standardized quality control remain essential requirements for future clinical application. Notably, few studies directly compare engineered EVs with alternative nanocarrier platforms, making it difficult to establish the true added value of EV-based delivery systems. Figure 4 summarizes the surface functionalization strategies employed to achieve disease-specific targeting in cardiovascular, neurological, cancer, and autoimmune diseases, while Table 5 provides a systematic overview of key studies.

**Table 5:** Summary of representative studies employing surface-functionalized EVs for therapeutic applications

Target diseases	EV source	Surface functionalization strategy	Targeting ligand	Therapeutic cargo	Outcomes	Reference
Cardiovascular diseases	Bone marrow MSCs	Cholesterol insertion	VEGF	SIRT1	Reduced inflammation and atherosclerosis	[164]
	Bone marrow MSCs	Genetic engineering	IMTP	-	Improved cardiac uptake	[165]
	Milk	Genetic engineering	IMTP	miR-30d	Reduced cardiac hypertrophy	[166]
	Cardiac Stem Cells	Click chemistry	CHP	-	Improved myocardial regeneration	[167]
	Blood	Click chemistry	Cardiac targeting peptide T	CRISPR-Cas9 RNP	Reduced myocardial damage	[156]
Neurological diseases	Neurons	Genetic engineering	F65 protein	Corynoxin-B	Reduced neuronal toxicity	[172]
	MSCs	Genetic engineering	RVG	-	Reduced inflammation in Alzheimer's disease	[173]
	HEK293T cells	Genetic engineering	RVG	DNA aptamer	Improved motor function in Parkinson's disease	[174]
	Adipose-derived stem cells	Click chemistry	Dopamine coating	-	Autophagy activation	[175]
	MSCs	Click chemistry	c(RGDyK)	Curcumin	Reduced inflammation and neuronal apoptosis	[157]
	M2 microglia	Hybrid coating	PDA + RVG29	miR-221-3p and miR-423-3p	Neuroprotection and reduced ischemic damage	[176]
Cancer diseases	T-cells	Genetic engineering	IL2	miR-181a-3p and 223-3p	Enhanced CD8 + activity and reduced PDL1	[179]
	Bone marrow MSCs	Genetic engineering	Targeting peptide of integrin α	miR-148a-3p	Reduced pancreatic tumor growth	[180]
	Bone marrow MSCs	Genetic engineering	Targeting peptide of integrin α	miR-138-5p and PFD	CAF reprogramming and tumor suppression	[181]
	Bone marrow MSCs	Genetic engineering	HSSP	TMZ	Glioblastoma tumor suppression	[143]
	Orange	Genetic engineering	RGD	siRNA anti-STAT3	Reduced ovarian cancer growth	[183]
	Grapefruit	Protein coating	LF	PS	Reduced cell migration and viability in lung cancer	[184]
Autoimmune diseases	MSCs	Membrane disruption	LJM-3064 aptamer	-	Remyelination in MS	[187]
	MSCs	Click chemistry	HA-PEG	-	Improved targeting inflamed tissue	[188]
	Grapefruit + MSC	Fusion engineering	CCR6	CX5461	Reduced inflammation in psoriasis	[189]

*Abbreviations*: *EVs* extracellular vesicles, *MSCs* mesenchymal stem cells, *VEGF* Vascular Endothelial Growth Factor, *SIRT1* silent information regulator 2–related enzyme 1, *IMTP* Ischemic Myocardium Targeting Peptide, *miR* micro RNA, *CHP* Cardiac Homing Peptide, *CRISPR* Clustered Regularly Interspaced Short Palindromic Repeats, *RNP* ribnucleoprotein, *RVG* Rabies Viral Glycoprotein, *HEK* human embrionyc kidney, *RGD* integrins targeting peptide, *PDA* Polydopamine, *IL2* Interleukin 2, *PFD* pirfenidone, *CAF* cancer-associated fibroblast, *HSSP* HMOX1-specific peptide, *TMZ* temozolomide, *siRNA* small interfering RNA, *LF* Lactoferrin, *PS* pterostilbene, *MS* multiple sclerosis, *HA-PEG* hyaluronic acid-Polyethylene Glycol, *CCR6* Chemokine Receptor Type 6

## Challenges, clinical translation and future perspectives

Due to their high biocompatibility, low immunogenicity, and unique ability to transfer macromolecules across biological barriers, EVs have attracted increasing interest as therapeutic delivery systems compared to synthetic nanocarriers. Advances in cargo loading and surface engineering strategies have enhanced the potential of EVs as therapeutic delivery vehicles. EV surface modifications with peptides, antibodies, and other bioactive molecules can improve EV-specific targeting, reducing off-target biodistribution. In this review, we have summarized and discussed multiple studies, demonstrating the application of surface-functionalized EVs in the treatment of several diseases, highlighting their therapeutic potential. Nevertheless, the clinical translation of functionalized EVs remains limited by multiple interconnected issues. These barriers fall into four main categories: scalable manufacturing, quality control and standardization, biological performance, and regulatory requirements.

The first challenge for the clinical translation of engineered EVs concerns the establishment of reproducible and scalable manufacturing processes. The production of clinical-grade EVs requires tight control over every step of the manufacturing workflow, from donor cell selection to EV isolation and batch release. In this regard, a major limitation is the absence of a standardized protocol for EV isolation, which significantly impacts scalable production and reproducibility across studies [190]. A major source of variability originates from donor cells themselves. Indeed, EV composition is influenced by cell culture conditions, cell donor type, and cell state. Consequently, variation in EV cargo and composition may lead to unpredictable biological responses in recipient cells, making it difficult to obtain consistent therapeutic products. This is exactly why donor cell selection represents a critical determinant of EVs for drug delivery. Parameters such as donor variability, cell passage number, and cell culture conditions can significantly influence EV yield and molecular profile. Therefore, defining standardized criteria for donor cells and determining controlled manufacturing conditions will be essential to ensure reproducibility and safety of EV-based therapies.

Even when donor cell-related variability is minimized, the absence of standardized isolation methods further complicates manufacturing. Different isolation methods may affect EV yield, composition, and purity. Taken together, these issues further contribute to batch-to-batch variability, one of the biggest obstacles preventing large-scale production of EV-based therapeutics. As a result, different batches of the same EV product could elicit opposing effects, compromising patient safety and the therapeutic outcome. Batch-to-batch variability is further related to differences in purification strategies and storage conditions. For instance, small changes in centrifugation parameters or filtration steps can affect EV purity. Furthermore, engineered EV preparations must be cleared of residual drugs or ligands, or co-isolated contaminants (aggregates, proteins, and lipids). Incomplete removal of these components may compromise purity and safety of the preparation [191]. In addition, freeze–thaw cycles or prolonged storage may alter EV stability and composition. Particularly, long-term preservation may induce cargo degradation and structural changes, affecting EV size and integrity, thereby influencing experimental reproducibility and product quality. Although recent studies have identified several new buffers to preserve EV integrity in prolonged storage [192, 193] no consensus has been reached on the optimal storage conditions. In addition, lyophilization has recently emerged as an alternative to conventional cryopreservation, potentially enabling long-term storage, simplifying transport, and reducing storage costs. However, recent studies observed alterations in EVs after lyophilization, highlighting the needed of standardized procedures to maximize EV preservation [194]. Another important aspect is the scalability of EV production, which, depending on living cells, is highly complex and costly. Indeed, expanding cell cultures while maintaining stable EV composition remains challenging, particularly when clinical-scale quantities are required. Bioreactor-based systems and optimized cell culture platforms may represent promising solutions to increase EV yield; however, these systems require further validation [195]. Furthermore, the manufacturing process must comply with stringent Good Manufacturing Practice (GMP) requirements, ensuring process reproducibility. However, implementing GMP-compliant production of engineered EVs remains challenging due to their high heterogeneity, which, combined with variations in manufacturing parameters, may significantly affect the final product. Furthermore, economic feasibility remains a critical consideration, as large-scale EV production requires expensive cell culture systems, purification platforms, and extensive quality testing. Reducing production costs while maintaining product consistency will be essential for clinical translation.

Beyond manufacturing, ensuring the quality and consistency of the final product represents another fundamental requirement. In this context, comprehensive product quality assessment -including EV characterization, quality control procedures, and potency assays- is a major bottleneck. EV characterization, which is crucial for accurate EV quantification and to establish the presence of EVs, is challenged by their small size and heterogeneity. Consequently, no single method can fully satisfy EV characterization needs, and employing orthogonal methods is recommended [26]. Moreover, characterization should not only confirm EV presence but also provide information on key parameters including particle concentration, size distribution, surface marker expression, and cargo composition. However, the lack of universally accepted criteria to define EV identity remains a major obstacle. Beyond characterization, the establishment of standardized quality control procedures and potency assays represents a fundamental requirement to support the regulatory evaluation for clinical translation. Quality control should verify purity, size distribution, surface markers of engineered EVs, as well as the absence of residual contaminants. In addition, potency assays are needed to verify the biological activity of engineered EVs. Unlike conventional analytical assays, which mainly assess product composition, potency assays aim to determine the functional effect of EV preparations. These assays should include cellular uptake, cargo delivery efficiency, immunomodulatory activity, and tissue-specific responses. However, the development of standardized potency assays remains a major challenge for clinical translation, as no universally functional test is available to predict the therapeutic efficacy of EV preparations. These limitations represent a barrier to regulatory approval, as well-defined product characteristics, validated manufacturing processes, and quality attributes are required. Currently, the lack of internationally accepted standards for EV-based therapies complicates the establishment of a regulatory framework. Therefore, standardized manufacturing, robust potency assays, and quality control will be essential to support product release and regulatory approval during clinical translation.

Even after successful manufacturing and quality assessment, several biological challenges still limit the therapeutic performance of engineered EVs following systemic administration. A major limitation is the limited understanding of EV in vivo behaviour, including biodistribution and pharmacokinetics. Repeated systemic administration may induce potential immunogenic responses, activating rapid clearance by the mononuclear phagocyte system [196]. This treatment-induced immune response represents an obstacle to EV distribution in vivo, reducing therapeutic efficiency [197]. Although surface engineering is designed to enhance tissue targeting, EV modification may also influence circulation time and immune recognition. Comprehensive pharmacokinetics studies, particularly after repeated administration, are required to optimize dosing strategies.

In this context, emerging artificial intelligence (AI) approaches offer promising opportunities to address several of these limitations [198]. AI-driven strategies are increasingly being applied to nanoparticle design and drug delivery optimization and could be leveraged in the EV field to guide surface engineering, optimize ligand selection, and improve cargo loading strategies. Furthermore, AI models may assist in predicting EV biodistribution, cellular uptake, and therapeutic response, as well as in identifying optimal biomarkers and target cells [199, 200]. Integrating AI with high-throughput experimental data could also facilitate the development of standardized and reproducible workflows, ultimately supporting quality-by-design approaches for EV-based therapeutics [80]. Future research should focus on the development of standardized workflows for EV isolation and characterization, as well as the enhancement of loading efficiency and in vivo stability. In parallel, addressing large-scale production, process reproducibility, and cost-effectiveness will be essential to enable clinical translation [195]. In this context, plant-derived EVs have emerged as a promising alternative, offering potential advantages in terms of scalability, low-cost production, and reduced immunogenicity for large-scale applications [201]. Addressing these biological and manufacturing limitations will be essential to realize the potential of EVs in therapeutic drug delivery.

## Data Availability

Not applicable.

## Abbreviations

AML: Acute myeloid leukemia
APP: Amyloid-β precursor protein
ASOs: Antisense oligonucleotides
BMSC: Bone marrow mesenchymal stem cell
BBB: Blood-brain barrier
CAP: Chondrocyte-affinity peptide
CML: Chronic myeloid leukemia
CME: Clathrin-mediated endocytosis
CVD: Cardiovascular diseases
DTX: Docetaxel
DOX: Doxorubicin
EVs: Extracellular vesicles
GEV: Grapefruit EVs
GBM: Glioblastoma
IMTP: Ischemic myocardium targeting peptide
ISEV: International Society of Extracellular Vesicles
LF: Lactoferrin
MISEV: Minimal Information for Studies of Extracellular Vesicles
MVB: Multivesicular bodies
MSC: Mesenchymal stem cell
MS: Multiple sclerosis
PDA: Polydopamine
PEG: Polyethylene glycol
PFD: Pirfenidone
PS: Pterostilbene
PDNVs: Plant derived nanovesicles
PDT: Photodynamic therapy
RBCEVs: Red blood cell-derived EVs
RGD: Integrins targeting peptide
RVG: Rabies viral glycoprotein peptide
ROS: Reactive oxygen species
SIRT1: Silent information regulator 2–related enzyme 1
GMP: Good Manufacturing Process
